# Functional Connectivity Changes Across the Spectrum of Subjective Cognitive Decline, Amnestic Mild Cognitive Impairment and Alzheimer’s Disease

**DOI:** 10.3389/fninf.2019.00026

**Published:** 2019-04-24

**Authors:** Ziqi Wang, Kaini Qiao, Guanqun Chen, Danyang Sui, Hao-Ming Dong, Yin-Shan Wang, Hui-Jie Li, Jie Lu, Xi-Nian Zuo, Ying Han

**Affiliations:** ^1^Department of Neurology, Xuanwu Hospital, Capital Medical University, Beijing, China; ^2^Department of Neurology, Chengdu Fifth People’s Hospital, Chengdu, China; ^3^CAS Key Laboratory of Behavioral Science, Institute of Psychology, Beijing, China; ^4^Research Center for Lifespan Development of Mind and Brain (CLIMB), Institute of Psychology, Beijing, China; ^5^Department of Psychology, University of Chinese Academy of Sciences (CAS), Beijing, China; ^6^Department of Radiology, Xuanwu Hospital, Capital Medical University, Beijing, China; ^7^Department of Nuclear Medicine, Xuanwu Hospital, Capital Medical University, Beijing, China; ^8^Beijing Key Laboratory of Magnetic Resonance Imaging and Brain Informatics, Beijing, China; ^9^Center of Alzheimer’s Disease, Beijing Institute for Brain Disorders, Beijing, China; ^10^Beijing Institute of Geriatrics, Beijing, China; ^11^National Clinical Research Center for Geriatric Disorders, Beijing, China

**Keywords:** network neuroscience, brain connectivity, centrality, Alzheimer’s disease, subjective cognitive decline, amnestic mild cognitive impairment

## Abstract

The abnormality occurs at molecular, cellular as well as network levels in patients with Alzheimer’s disease (AD) prior to diagnosis. Most previous connectivity studies were conducted at 1 out of 3 (local, meso and global) scales in subjects covering only part of the entire AD spectrum (subjective cognitive decline, SCD; amnestic mild cognitive impairment, aMCI; and then fully manifest AD). Data interpretation within the framework of disease progression is therefore difficult. The current study included 3 age- and sex-matched cohorts: SCD (*n* = 32), aMCI (*n* = 37) and fully-established AD (*n* = 30). A group of healthy elderly subjects (*n* = 40) were included as a normal control (NC). Network connectivity was examined at the local (degree centrality), meso [subgraph centrality (SC)], and global (eigenvector and page-rank centralities) levels. As compared to NC, SCD subjects had isolated decrease of SC in primary (somatomotor and visual) networks. aMCI subjects had decreased centralities at all three scales in associative (frontoparietal control, dorsal attention, limbic and default) networks. AD subjects had increased centrality at the global scale in all seven networks. There was a positive association between Montreal Cognitive Assessment (MoCA) scores and DC in the frontoparietal control network in SCD, a negative relationship between Mini-Mental State Examination (MMSE) scores and EC in the somatomotor network in AD. These findings suggest that the primary network is impaired as early as in SCD. Impairment in the associative network also starts at the local level at this stage and may contribute to the cognitive decline. As associative network impairment extends from local to meso and global scales in aMCI, compensatory mechanisms in the primary network are activated.

## Introduction

Brain pathology of Alzheimer’s disease (AD) occurs decades before the manifestations of clinical AD (Dubois et al., 2016). With the pathological cascade, three different stages show the progression of AD: preclinical AD, mild cognitive impairment (MCI) and late stage of AD (Sperling et al., 2011). Subjective cognitive decline (SCD) in the setting of preclinical AD is defined by self-perception of worsening cognitive capacity but no impairment in cognition on standard neuropsychological assessments and no evidence for MCI or prodromal AD or dementia (Jessen et al., 2014). SCD can significantly predict MCI or dementia (Rabin et al., 2017). MCI, especially amnestic mild cognitive impairment (aMCI), progresses to AD or other forms of dementia more than people without MCI (Kantarci et al., 2009). Such a three-stage continuum of AD progression (SCD, aMCI, and AD) offers us a systematic perspective to study AD.

Resting-state functional magnetic resonance imaging (rfMRI) has been increasingly used as a reliable method (Zuo and Xing, 2014) to detect brain network abnormalities in aMCI (Agosta et al., 2012; Bharath et al., 2017; Wang et al., 2018b) or AD (Binnewijzend et al., 2014; Wang et al., 2018b). The rfMRI findings in AD and MCI are rather consistent across different studies in different networks, such as default mode network (Agosta et al., 2012), somatomotor network (Albers et al., 2015), dorsal attention network (Qian et al., 2015), limbic network (Nestor et al., 2003), and frontoparietal control network (Agosta et al., 2012; Brier et al., 2012; Munro et al., 2015). Nevertheless, relatively few studies have been systematically examined across the entire three-stage continuum of AD progression. Furthermore, rare quantitative conclusions of brain network changes have been drawn on the dynamical mechanism of the disease deterioration. Meanwhile, from a methodological view, most previous studies focused on a single scale of functional brain organization in AD, e.g., only at the global scale (Supekar et al., 2008; Binnewijzend et al., 2014) or only at the local scale (Grady et al., 2003; Klaassens et al., 2017). Thus, an examination of multi-scale network topology across SCD, aMCI, and AD would enhance the current understanding of neuroimaging pathology of AD progression.

Network analyses of human brain functional connectomes, based on graph theory, can advance our understanding of the multi-scale intrinsic architecture of the human brain connectome using different centralities (Zuo et al., 2012). Degree centrality (DC) is the number of direct connections to a brain network node and reflects local-scale connectivity. Subgraph centrality (SC) characterizes the odd-cyclic subgraph or closed walk of the network node, and represents a connectivity measure at meso-scale (Zuo et al., 2012). Both eigenvector centrality (EC) and page-rank centrality (PC) determine the nodal connectivity with their adjacency connectivity at global-scale (Zuo et al., 2012). More information about DC, SC, EC, and PC can also be seen in Supplementary Materials 1. Recently, a few studies have applied network centrality at a single scale, such as EC (Binnewijzend et al., 2014; Adriaanse et al., 2016; Lou et al., 2016; Qiu et al., 2016) or DC (Guo et al., 2016) in MCI or AD.

In this study, we aimed to draw a full picture of functional changes by using network centrality at multi-scale (DC, SC, EC, PC) in AD continuum (SCD, aMCI, and AD) and age- and sex-matched healthy elderly subjects as normal control (NC). Additionally, we evaluated the relationship between network centrality at multi-scale and cognitive performances. Given the three stages of AD continuum, we hypothesized that a progression-dependent pattern of network centrality changes was detectable at multiple scales.

## Materials and Methods

### Participants

All our subjects are from the database (NCT 02353884, 02353845, 02225964). A total of 188 patients, including 47 SCD, 93 aMCI and 48 AD, were recruited from the memory clinic of neurology department of Xuanwu Hospital, Capital Medical University. While 92 NC were recruited by advertisement from the local community. All the subjects had no history of stroke, head injury, or other major neuropsychiatric illness, such as Parkinson’s disease, encephalitis, epilepsy, psychosis or congenital mental growth retardation, alcohol or drug abuse, and other diseases or treatments that can affect cognitive functions (Morris, 1993). After being age- and sex-matched for each group, 139 subjects (40 NC, 32 SCD, 37 aMCI, 30 AD) were included for final analysis.

### Demographic, Clinical, and Cognitive Variables

The diagnoses for SCD, aMCI, and AD were made in consensus by two consultant psychiatrists. The criteria for AD has been reported in detail in the previous study (Wang et al., 2014). Briefly, we diagnosed AD using the revised version of Diagnostic and Statistical Manual of Mental Disorders 4th Edition (DSM-IV) Criteria (American Psychiatric Association, 1994) for Dementia and the National Institute of Neurologic and Communicative Disorders and Stroke-Alzheimer’s Disease and Related Disorders Association (NINCDS-ADRDA) Criteria (McKhann et al., 1984) for possible or probable AD. In addition, patients with AD had the Clinical Dementia Rating scale (CDRs) score of 1 and were older than 50 years old (Morris, 1993). The criteria of aMCI was as follows: (1) memory complaint (if possible) confirmed by an informant; (2) preserved activities of daily living; (3) the scores for the Chinese version of the Mini-Mental State Examination (MMSE) ≥ 24; (4) CDRs score = 0.5 (Portet et al., 2006); (5) not demented according to the DSM-IV (Petersen et al., 1999, 2001; Petersen, 2003); and (6) age older than 50 years old. More information about the criteria of aMCI has been described in detail in a previous study (Zhang et al., 2017). The criteria of SCD (Shu et al., 2018) included: (1) self-reported persistent memory decline, which was confirmed by informants; (2) performing normally on the MMSE or the Beijing version of the Montreal Cognitive Assessment (MoCA; adjusted for age, sex, and education); (3) CDRs score = 0; and (4) age older than 50 years old. The criteria of NC were: (1) no self-reported persistent memory decline; (2) performing normally on the MMSE or MoCA (adjusted for age, sex, and education); (3) CDRs score = 0; and (4) age older than 50 years old.

We obtained information on age, sex and years of education *via* interview, and developed a standard clinical evaluation protocol as described above to collect scores for MMSE, Auditory Verbal Learning Test (AVLT), MoCA, and CDRs from all the participants.

### MRI Acquisition and Processing

Magnetic resonance imaging (MRI) scans were acquired at a 3.0 T Siemens scanner (Erlangen, Germany) at Beijing Xuanwu Hospital, Capital Medical University. Participants were all instructed to lie quietly and close their eyes, and received a T1-weighted structural MRI scan (MP-RAGE sequence: TR = 1,900 ms, TE = 2.2 ms, TI = 900 ms, FA = 9°, matrix = 256 × 256, slice thickness = 1.0 mm; 176 sagittal slices, no gap) and a rfMRI scan (EPI sequence: TR = 2,000 ms, TE = 40 ms, FA = 90°, 28 axial slices, 4 mm isotropic voxel, matrix = 64 × 64) of 8 min.

Both structural and functional image preprocessing were completed in the Connectome Computation System (CCS^1^), which has been described previously (Xu et al., 2015). Briefly, CCS extended the network centrality analyses (Zuo et al., 2012) from 3D volumetric element (voxel) to 2D surface element (vertex) by projecting the 3D rfMRI images onto 2D cortical surfaces (Chen et al., 2014). Such an analytic strategy has been demonstrated to be more effective to mitigate partial volume effects and increase test-retest reliability of rfMRI analyses (Zuo et al., 2013; Zuo and Xing, 2014). First, T1 images were employed to reconstruct individual cortical surfaces (Ségonne et al., 2004, 2007). Second, rfMRI images were preprocessed in individual native spaces to equilibrate, de-spike, correct slice time and motion, normalize global mean intensity, regress out the white matter, cerebrospinal fluid and Friston-24 motion parameters, band-pass (0.01–0.1 Hz) filter and remove linear and quadratic trends of the timeseries signals. Finally, the rfMRI images were matched to their individual structural images using a boundary-based registration (BBR) algorithm (Greve and Fischl, 2009). They were then further projected onto the *fsaverage5* cortical surfaces in the standard MNI space (10,242 vertices per hemisphere and 4 mm inter-vertex gap on average; Thomas Yeo et al., 2011).

Quality control procedure was carried out with CCS to high-quality preprocessed brain images for network centrality analysis. Specifically, screenshots were obtained for skull stripping, tissue segmentation, surface reconstruction, BBR image registration, and the head motion correction during rfMRI (Jiang et al., 2015). For those individuals with any of the first three showing bad quality, the brain extraction will be invented by manually editing. Meanwhile, head motion of each participant met following criterion: the mean frame-wise displacement (meanFD) < 0.2 mm, the maximum degree of rotational movement (maxRot) ≤ 2° and the maximum distance of translational movement (maxTran) ≤ 2 mm.

### Network Centrality Mapping and Statistics

The procedure of mapping the centrality metrics for individual functional connectomes completely followed the methods described by Zuo et al. (2012), except that the connectomes were constructed on cortical surfaces. The *fsaverage5* cortical surface meshes consisted of 17,064 vertices with the preprocessed rfMRI time series. Fisher-z transformed Pearson’s correlations were calculated between each paired vertices. The significance above the threshold (*p* = 0.0001, uncorrected) was used to determine an edge connecting. This generated individual binary adjacency matrices for subsequent network centrality computation. Specifically, given a node, its degree centrality (DC) was computed as the number of the edges connecting to the node, and commonly measured a nodal direct connectivity at a local network scale. SC measures the participation of a node in all subgraphs at a meso network scale and is calculated based on the first 20 eigenvalues and eigenvectors of the adjacency matrix. At a global network scale, eigenvector centrality (EC), which is the first eigenvector of the adjacency matrix, is the one that corresponds to the largest eigenvalue and can measure global features of the graph. PC is a variant EC and introduces a small probability of 0.15 for random damping to handle walking traps on a graph. All these four metrics of network centrality have been shown with moderate to high test-retest reliability in 3D volume space and should be more reliable for their versions of 2D surface space as computed in the present work, due to the previous observation on the reliability improvement of local functional connectivity with updates of computational space (Zuo et al., 2013).

For each of the four types of network centrality described above, its full cortical maps were first adjusted by individual intracranial volume and then fed the subsequent FreeSurfer group analysis to evaluate various group-level statistics. A FSGD (FreeSurfer Group Descriptor) file was constructed for the four groups of participants (NC, SCD, aMCI, and AD) to implement a set of ANCOVA using general linear models that considered diagnosis, sex, age, and years of education as covariates with three contrasts of group comparisons (SCD vs. NC, aMCI vs. NC, AD vs. NC). The vertex-wise significance values for each contrast of the group comparisons were corrected with false discovery rate (FDR) method (corrected *p* = 0.05, minimal surface cluster area = 25 mm^2^). The partial correlations between the mean centrality at cluster-level within most abnormal topology metrics (≥ 2 stages of SCD, aMCI, and AD) and behavioral measurements (MMSE, AVLT, MoCA) were also evaluated after controlling age, sex, and years of education. We used the Bonferroni corrections for multiple comparisons at *P* < 0.05 and for groups at all three scales.

**Figure 1:**
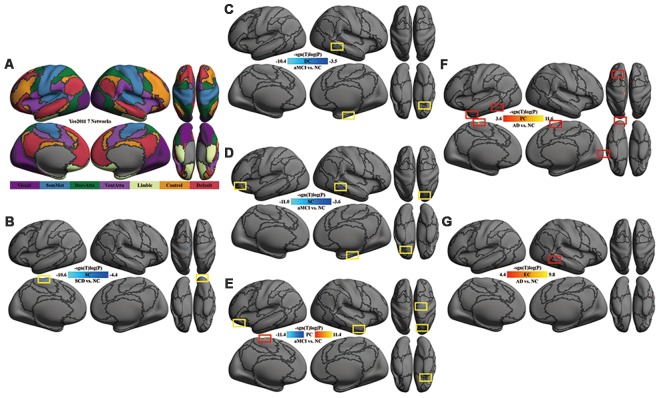
Multi-scale network centrality changes in AD progression. **(A)** The seven networks map in Yeo et al. (2011). **(B)** SC in SCD vs. NC. The reduced SC in SCD was located in the left somatomotor network (left paracentral cortex) and the right visual network (right occipital cortex). **(C)** DC in aMCI vs. NC. The reduced DC in aMCI were located in the right default network (orbital gyri), the right limbic network (parahippocampal areas) and the right frontoparietal control network (middle temporal area). **(D)** SC in aMCI vs. NC. Except for the default, limbic and frontoparietal control network, the reduced SC in aMCI extended to the left default network (orbital cortex) and the right dorsal attention network (superior parietal areas). **(E)** PC in aMCI vs. NC. Significant decreases of PC in aMCI were observed within the default network (right middle temporal and bilateral orbital gyrus), the right somatomotor network (precentral area), the right dorsal attention network (superior parietal area) and the left limbic network (orbital areas), whereas significant increases of PC in aMCI were detectable within the left somatomotor network (paracentral area). **(F)** PC in AD vs. NC. The increased PC in AD were located in the visual network (occipital areas), the left somatomotor network (paracentral area), the left limbic network (temporal pole), the left dorsal attention network (inferior temporal area), the left default network (superior frontal gyrus) and the right ventral attention network (superior frontal area). **(G)** EC in AD vs. NC. A significant increase of EC was found in the right frontoparietal control network (inferior temporal sulcus). All the above tests were thresholded at an false discovery rate (FDR) corrected significance level of *p* < 0.05. Gray curves indicate the boundaries according to the seven networks map in Yeo et al. (2011). Abbreviations: NC = normal control; SCD = subjective cognitive decline; aMCI = amnestic mild cognitive impairment; AD = Alzheimer’s disease; DC = degree centrality; SC = subgraph centrality; PC = page-rank centrality; EC = eigenvector centrality.

For the purpose of locating the network at both network-level and area-level, we reported the results with brain regions showing significant changes across the groups using the cortical parcellation of both functional networks (see Figure 1A), derived by Yeo et al. (2011) and anatomical Destrieux Atlas derived by Fischl et al. (2004).

## Results

Age, sex and years of education were well matched among groups. There were significant differences in MMSE, AVLT and MoCA scores among groups (*p* < 0.001). AD had significantly lower scores than the other three groups by subgroups analyzed (Table 1).

**Table 1:** Demographic information and behavioral measurements.

	NC (40)	SCD (32)	aMCI (37)	AD (30)	*F*/Chi-square	
Age (Years)	68.07 ± 6.44	66.70 ± 5.98	69.67 ± 7.48	69.61 ± 9.53	1.213	0.307
Sex (M/F)	16/24	12/20	19/18	12/18	1.693	0.638
Education (Years)	12.00 ± 4.44	11.59 ± 4.06	11.22 ± 3.97	10.03 ± 4.71	1.276	0.285
MMSE	28.93 ± 1.27_a_	27.53 ± 1.90_b_	26.49 ± 1.68_b_	17.27 ± 5.19_c_	113.595	<0.001
AVLT						
Immediate recall	9.15 ± 1.82_a_	7.74 ± 1.77_b_	6.12 ± 1.82_c_	3.78 ± 1.29_d_	57.210	<0.001
AVLT						
Delayed recall	9.85 ± 2.77_a_	8.28 ± 2.69_a_	4.27 ± 238_b_	1.04 ± 1.56_c_	75.514	<0.001
AVLT						
Recognition	12.03 ± 2.69_a_	10.50 ± 2.63_a_	8.35 ± 3.61_b_	4.41 ± 2.85_c_	37.825	<0.001
MoCA	27.06 ± 1.98_a_	26.24 ± 1.74_a_	20.80 ± 3.56_b_	13.07 ± 4.78_c_	109.004	<0.001

*Differences among diagnosis categories (NC, SCD, aMCI and AD) were tested with ANOVAs (LSD or Dunnett’s T3 post hoc comparisons; p < 0.05). Each subscript letter denotes a subset of diagnosis categories whose column proportions do not differ significantly from each other at the 0.05 level. Abbreviations: NC, normal control; SCD, subjective cognitive decline; aMCI, amnestic Mild Cognitive Impairment; AD, Alzheimer’s disease; MMSE, Mini-Mental State Examination; AVLT, Auditory Verbal Learning Test; MoCA, Montreal Cognitive Assessment*.

### Meso-scale Network Centrality Reduced in SCD

Compared with NC, SC was decreased in the left somatomotor network (paracentral cortex) and the right visual network (occipital cortex) in SCD patients (Table 2, Figure 1B).

**Table 2:** Full list of brain regions with significant SC differences between SCD and NC.

Contrast	Index	Hemi	Brain regions	Max (-log10p)	Size (mm^2^)	*X*	*Y*	*Z*	NV
SCD < NC	SC	LH	G_and_S_paracentral	−10.643	63.72	−6.9	−22.9	68.2	12
		RH	Pole_occipital	−7.409	91.49	20.2	−98.0	−2.9	7

*Abbreviations: SCD, subjective cognitive decline; NC, normal control; SC, subgraph centrality; LH, left hemisphere; RH, right hemisphere; X, Y, Z, coordinates of primary peak locations in the Talairach space; NV, number of vertex*.

### Multi-scale Network Centrality Altered in aMCI

Compared with NC, DC was decreased in the right default network (orbital gyri), the limbic network (left orbital and right parahippocampal areas) and the right frontoparietal control network (middle temporal area) in aMCI (Figure 1C). In aMCI, SC was decreased in the limbic network (left orbital cortex and right parahippocampal area), the left default network (the orbital cortex), the right dorsal attention network (superior parietal areas) and the right frontoparietal control network (middle temporal area; Figure 1D). In aMCI, PC was decreased in the default network (bilateral orbital gyrus, right middle temporal and left frontal areas), the right somatomotor network (precentral area), the right dorsal attention network (superior parietal area) and the bilateral limbic network (orbital areas), whereas, it was increased in the left somatomotor network (paracentral area; Table 3, Figure 1E).

**Table 3:** Full list of brain regions with significant centralities differences between aMCI and NC.

Contrast	Index	Hemi	Brain regions	Max (-log10p)	Size (mm^2^)	*X*	*Y*	*Z*	NV
aMCI < NC	DC	LH	G_orbital	−10.453	29.37	−24.8	11.6	−16.7	5
		RH	G_temporal_middle	−10.045	119.04	60.7	−38.4	−9.8	10
			G_orbital	−7.971	36.91	29.2	19.4	−18.4	6
			G_oc-temp_med-Parahip	−7.005	47.24	23.1	−19.3	−22.6	7
	SC	LH	G_orbital	−11.003	39.17	−33.6	25.0	−16.9	4
			G_orbital	−9.343	34.14	−24.8	11.6	−16.7	6
			G_orbital	−6.219	44.71	−43.7	35.9	−14.4	7
		RH	G_parietal_sup	−8.546	47.68	16.6	−67.5	45.7	5
			G_temporal_middle	−7.911	69.76	62.7	−36.0	−9.6	6
			G_oc-temp_med-Parahip	−5.842	40.90	25.1	−20.0	−18.9	6
	PC	LH	G_orbital	−10.316	78.77	−33.6	25.0	−16.9	8
			G_front_middle	−6.922	97.50	−22.3	59.7	5.6	7
		RH	G_orbital	−11.358	44.44	29.2	19.4	−18.4	7
			G_precentral	−10.918	47.73	18.6	−13.7	64.9	7
			G_parietal_sup	−10.064	47.68	16.6	−67.5	45.7	5
			G_temporal_middle	−8.712	60.58	54.5	0.1	−26.2	5
aMCI > NC	PC	LH	G_and_S_paracentral	10.152	33.13	−7.2	−20.0	68.4	6

*Abbreviations: aMCI, amnestic Mild Cognitive Impairment; NC, normal control; DC, degree centrality; SC, subgraph centrality; PC, page-rank centrality; LH, left hemisphere; RH, right hemisphere; X, Y, Z, coordinates of primary peak locations in the Talairach space; NV, number of vertex*.

### Global-Scale Network Centrality Enhanced in AD

Compared with NC, AD had an increase of global network centrality but lacked any centrality changes at both local and meso scales (Table 4). PC was increased in the visual network (occipital areas), the left somatomotor network (paracentral area), the left limbic network (temporal pole), the left dorsal attention network (inferior temporal area), the left default network (superior frontal gyrus) and the right ventral attention network (superior frontal area; Figure 1F). EC was increased in the right frontoparietal control network (inferior temporal sulcus; Figure 1G).

**Table 4:** Full list of brain regions with significant centralities differences between AD and NC.

Contrast	Index	Hemi	Brain regions	Max (-log10p)	Size (mm^2^)	*X*	*Y*	*Z*	NV
AD > NC	PC	LH	G_and_S_paracentral	11.596	87.73	−6.9	−22.9	68.2	16
			Pole_occipital	11.142	90.27	−13.3	−99.4	5.4	8
			Pole_temporal	10.471	85.61	−41.9	−0.1	−29.4	6
			G_temporal_inf	8.850	99.45	−50.8	−45.8	−14.6	7
			S_front_sup	4.067	80.70	−31.7	14.7	43.2	7
		RH	Pole_occipital	11.211	284.74	20.2	−98.0	−2.9	22
			G_front_sup	8.651	56.65	10.7	4.5	61.3	8
	EC	RH	S_temporal_inf	9.807	90.48	56.8	−44.2	−10.8	7

*Abbreviations: AD, Alzheimer’s disease; NC, normal control; PC, page-rank centrality; EC, eigenvector centrality; LH, left hemisphere; RH, right hemisphere; X, Y, Z, coordinates of primary peak locations in the Talairach space; NV, number of vertex*.

### *Post hoc* Cluster-Level Analyses

**Figure 2:**
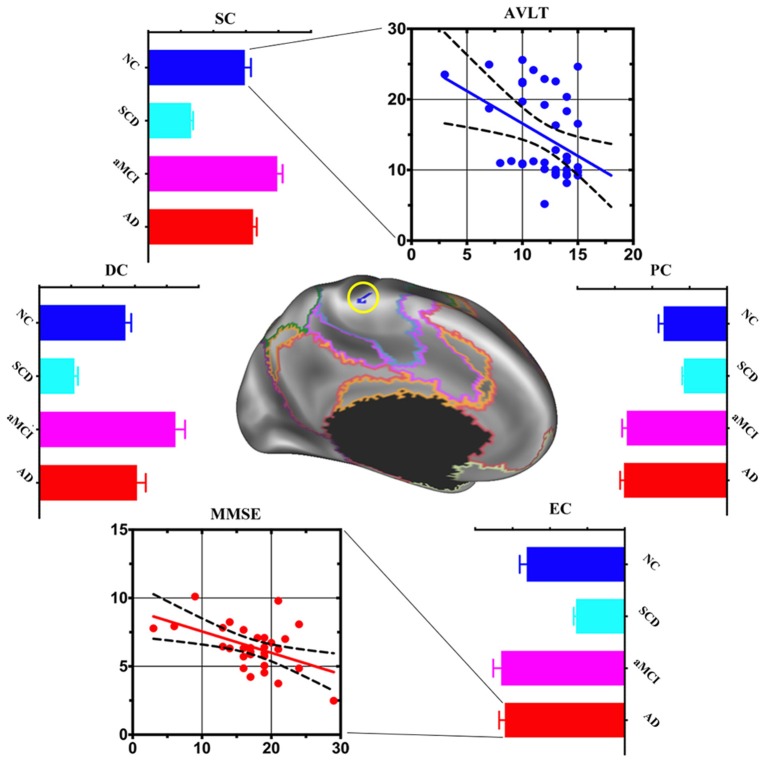
*Post hoc* cluster-level analyses in the left somatomotor network. Multi-scale centrality and behavioral performance in the left somatomotor network region among NC, SCD, aMCI and AD. Compared with NC, the area of the left somatomotor network showed centralities change at local, meso and global scales in AD progression. Mean DC, SC and EC values decreased in SCD, increased in aMCI and AD. The scatter plot exhibited negative association between: (1) mean SC values and auditory verbal learning test (AVLT)-Recognition scores in the NC (*r* = −0.4093, *p* < 0.05); and (2) mean EC values and Mini-Mental State Examination (MMSE) total scores in the AD (*r* = −0.4908, *p* < 0.05).

Compared with NC, the area of the left somatomotor network showed changes in centralities at local, meso and global scales in AD progression. DC, SC, PC and EC decreased in SCD but increased in aMCI and AD. When the relationship between the centralities and cognitive performance was deeply analyzed, negative associations between SC and AVLT-Recognition scores in NC (*r* = −0.4093, *p* < 0.05) and between EC and MMSE total scores in AD (*r* = −0.4908, *p* < 0.05) were found (Figure 2).

**Figure 3:**
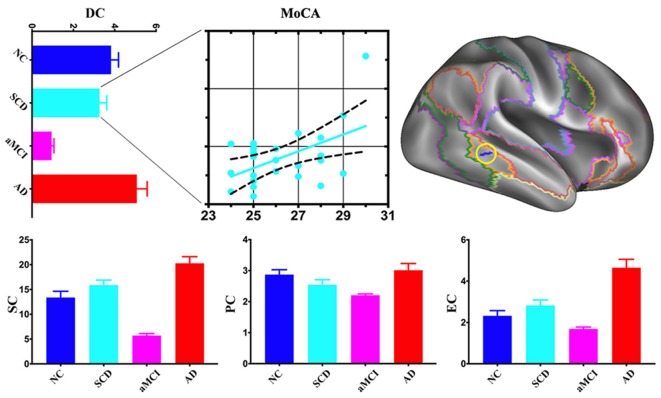
*Post hoc* cluster-level analyses in the right frontoparietal control network. aMCI showed the lowest centralities at local, meso and global scales in the area of the right frontoparietal control network. The scatter plot exhibited a positive association between mean DC values and Montreal Cognitive Assessment (MoCA) total scores in the SCD (*r* = 0.5076, *p* < 0.05).

Compared with NC, the area of the right frontoparietal control network also exhibited multi-scale network centrality changes in AD progress. SCD had a decrease of DC and PC and an increase of SC and EC. DC, SC, PC, and EC decreased in aMCI but increased in AD. SCD group showed a significant positive association between DC and MoCA scores (Figure 3).

## Discussion

The main finding of the present study is that different functional network centralities changed at different scale levels across the spectrum of SCD, aMCI, and AD. aMCI exhibited multi-scale abnormal centralities, while SCD and AD exhibited single-scale abnormal centrality: (1) primary, meso-scale, and impairment in SCD; (2) both primary and associative, impairment and compensation coexisted in aMCI; and (3) both primary and associative, extended global compensation in widespread networks in AD.

### Meso-scale Topological Impairment in Primary Network in SCD

In this study, we observed decreased meso centrality (SC) in the left somatomotor network and right visual network in individuals with SCD. It is worth noting that we found no enhanced centralities in SCD. Compensatory mechanisms, frequently proposed in aMCI (Qi et al., 2010) and AD (Agosta et al., 2012), seem to have not yet happened at this point since brain network impairments are not yet severe enough. The motor system receives sensory information for movement control (Rizzolatti et al., 1997). Many pyramidal and extrapyramidal motor impairments affect a substantial portion of AD patients and progressively worsen along with cognitive impairment (Albers et al., 2015). The onset of accelerated rates of motor decline can occur 12 years before the onset of MCI in initially cognitively healthy adults (Buracchio et al., 2010). In the present study, the decrement of multi-scale centrality in the somatomotor network may indicate motor dysfunction and further supports the theory that motor impairment could occur at an early stage of AD, or even precede the onset of the cognitive impairment for AD by a decade and longer (Albers et al., 2015). The previous study has detected an increased functional brain network efficiency during the audiovisual task in aging (Wang et al., 2018a), while there is a negative connection between within-network functional connectivity in the visual network and levels of SCD (Contreras et al., 2017). These results indicate visual network impairment beginning from SCD. Our result provides further evidence for topological impairment in the visual network, which may be associated with early indications of cognitive impairment. In summary, these findings might help us to better identify or understand early, multi-scale primary network (e.g., sensory and motor) impairments caused by the early AD.

### Multi-scale Topological Impairment and Compensation Activated in aMCI

Global-scale centrality impairment and compensation in primary network coexist in aMCI. We found both increased and decreased PC in aMCI in the somatomotor network. Earlier studies have reported both increased and decreased brain connectivity in aMCI as well (Qi et al., 2010; Wang et al., 2015). In contrast to SCD with only decreased centrality at the meso scale and AD with only increased centrality at global-scale, aMCI exhibited bidirectional alterations of brain network centrality at the global-scale. Summarizing the content, we draw a conclusion that disconnection syndrome (Qiu et al., 2016) and compensation in primary network coexist in aMCI.

Multi-scale centrality impairment in associative networks occurs in aMCI. We found decreased DC, SC, and PC in the limbic and default mode network, reduced SC and PC in the dorsal attention network, as well as declined DC and SC in the frontoparietal control network in aMCI. In previous studies, atrophy (Callen et al., 2001) and hypometabolism (Nestor et al., 2003) in the limbic network in AD have been widely reported. Brain alterations in the default mode network in aMCI, such as amyloid deposition (Agosta et al., 2012), atrophy (Thompson et al., 2003), decreased activity (Sorg et al., 2007), and reduced connectivity (Qi et al., 2010) have also been reported. Our findings in the two networks (limbic and default) are consistent with previous studies and add the evidence for functional disconnection in aMCI. In the present study, significant meso- and global-scale topological impairments were found in the dorsal attention network but not in the ventral attention network. These findings suggest that functional connectivity appears to be preferentially affected in the dorsal attention network and preserved or less impaired in the ventral attention network in aMCI (Sorg et al., 2007; Qian et al., 2015). Dorsal attention network is involved in the endogenous attention orienting (top-down) process (Fox et al., 2006), while ventral attention network is responsible for reorienting attention in response to salient sensory stimuli (Fox et al., 2006; bottom-up process). In aMCI patients, deteriorations in goal-relevant processing such as divided attention and selective attention (Dannhauser et al., 2005; Redel et al., 2010) have occurred, while still retain the ability for bottom-up processing (Zhang et al., 2015). This asymmetric pattern of network topology impairments of attention networks might help us better understand attention deficits in patients with aMCI. As for the frontoparietal control network, previous studies are not quite consistent. One study reported decreased connectivity in aMCI (Munro et al., 2015), while another one reported increased connectivity (Agosta et al., 2012). The discrepancy between these studies may be attributed to differences in severity of cognitive impairment and diagnostic criteria for patients. Centrality alterations, at the local and meso rather than global scales in our study, may suggest relatively less impairment in the frontoparietal control network in the stage of aMCI.

### Global Compensation in All Seven Networks in AD

An intriguing finding of this study is that we probe a unique pattern of compensation in AD patients: enhanced global centrality in large scale was observed in all seven networks (both primary and associative networks). This result is consistent with previous studies, which revealed increased activity and connectivity in AD (Zhou et al., 2010; Agosta et al., 2012). A possible reason for such augments in AD may be that additional neural resources are recruited to compensate for losses. And this hypothesis has been supported by earlier studies showing that patients with AD are able to succeed in episodic memory tasks due to compensatory neuronal activity (Buckner, 2004; Schwindt and Black, 2009). There is an alternate network, a compensation network, consisting of the left posterior temporal cortex, calcarine cortex, posterior cingulate, and the vermis (Stern et al., 2000). Our study showed that centrality enhanced at the global scale in AD, which suggests that compensation in this stage of the disease has extended from local to remote. Furthermore, compensation is also active in both primary and associative networks.

### Progressed From Local to Global, Impairment to Compensation in AD Continuum

Our previous study showed that the rich club of the human connectome was disrupted from SCD to AD (Yan et al., 2018). In the current study, SCD exhibited only primary network (sensory and motor) impairments, while aMCI and AD progressed to associative network impairments, such as limbic, default, attention and frontoparietal control networks. In addition, SCD displayed meso impairment, aMCI demonstrated local, meso and global scale alterations (impairment and compensation coexist), but AD had only global compensation. These findings show a progressive pattern of functional brain network in AD continuum: impairment occurs as early as in SCD (decreased SC) and continues and becomes severe enough in aMCI, then compensation is warranted.

When focused on both time and spatial cluster-level analysis, two interesting areas were found. In the left somatomotor network, centrality at all three levels decreased in SCD but increased in aMCI and AD. In addition, augmented centrality at global-scale only in AD exhibited a significantly negative relationship with cognitive performance (Figure 2). These findings provide evidence that compensatory mechanisms followed with clinical mechanisms progressed. As to the right frontoparietal control network (Figure 3), centralities decreased at the local scale, increased at the meso scale and coexisted at the global scale in SCD, while they decreased in aMCI and increased in AD at all three levels. Furthermore, only decreased centrality at local-scale in SCD showed a significant positive association with cognitive performance. We proposed that local associative network impairment directly affected cognitive function at the very early stage of AD, but subtle compensatory function at the meso and global scale balanced further cognitive impairment.

Based on the results from the current study, we hypothesize that brain network impairment starts in the primary network in SCD. Impairment in the associative network also starts at the local level at this stage and may contribute to the cognitive decline. As associative network impairment extends from local to meso and global scales in aMCI, compensatory mechanisms in the primary network are activated. Such a progressive pattern across the spectrum of SCD, aMCI, and AD, may underlie increased network topological scale and gives a dynamical description of the pathology of AD progression.

### Limitations

Several limitations should be mentioned here: first, our study was not a real cohort, a longitudinal design in the future would still be necessary to quantitatively elucidate its dynamic topological changes. Second, we only had resting state functional magnetic resonance imaging (fMRI) data for this study, adding biomarkers will be more persuasive. Third, the fMRI data sets in this study had limited spatial and temporal resolutions, better spatial-temporal resolutions would definitely strengthen our conclusion.

## Conclusion

SCD had an isolated decrease of SC in the primary (somatomotor and visual) networks. aMCI had both a decrease and an increase of global centrality in the primary motor network, as well as decreases at all three levels in associative (frontoparietal control, attention, limbic and default) network areas. AD had increased centrality at the global scale in all seven networks. In the cluster level, brain network impairment starts in the primary network in SCD. Impairment in the associative network also starts at the local level at this stage and may contribute to the cognitive decline. As associative network impairment extends from local to meso and global scales in aMCI, compensatory mechanisms in the primary network are activated. Such a progressive pattern across the spectrum of SCD, aMCI, and AD, may underlie increased network topological scale and gives a dynamical description on the pathology of AD progression.

## Ethics Statement

All our subjects are from our database (NCT 02353884, 02353845, 02225964). This study was approved by the medical research ethics committee and institutional review board of Xuanwu Hospital, Capital Medical University. Written informed consents were obtained from all the participants and/or at least one of their families prior to the data acquisition.

## Author Contributions

All authors listed have made a substantial, direct and intellectual contribution to the work, and approved it for publication.

## Conflict of Interest Statement

The authors declare that the research was conducted in the absence of any commercial or financial relationships that could be construed as a potential conflict of interest.

## Footnotes

^1^http://lfcd.psych.ac.cn/ccs.html
