# Supplementary material for: Functional Connectivity Changes Across the Spectrum of Subjective Cognitive Decline, Amnestic Mild Cognitive Impairment and Alzheimer’s Disease
Source: Front Neuroinform. 2019 Apr 24;13:26. doi: 10.3389/fninf.2019.00026 (PMC6491896; doi:10.3389/fninf.2019.00026)
Supplement: Supplementary file 1 [file Data_Sheet_1.docx]

Supplementary materials 1

*Centrality Indices*

Since a graph had been abstracted and formatted from data, we constructed graphs at voxel level. Then we calculated the temporal Pearson’s correlation ($r_{ij}$) of times series between the *i-* and *j-*th voxels and constructed a correlation matrix R = ($r_{ij}$), 1≤*i*, *j*≤N (N is the number of voxels). Next, we used *P* = 0.0001 (uncorrected) as a statistical significance threshold of correlation $r_{0}$. Finally, we set up an adjacency matrix $A = {(a_{ij})}_{1\leq i\leq N,1\leq j\leq N}$ of a binary graph (1a) or a weighted graph (1b) as following:

$a_{ij}= \left\{ \begin{aligned} 0,r_{ij}<r_{0} \\ 1,r_{ij}\geq r_{0} \end{aligned} \right.$ (1a)

$a_{ij}= \left\{ \begin{aligned} 0,r_{ij}<r_{0} \\ r_{ij},r_{ij}\geq r_{0} \end{aligned} \right.$ (1b)

1. *Degree Centrality (DC)*

DC, the most local centrality measure, is computed as in the following equation:

$$\mathrm{DC}\left( i \right)=\sum_{j=1}^{N} a_{ij}$$

1. *Subgraph Centrality (SC)*

A network comprises of subgraphs and SC is used to measure the participation of a node in all subgraphs (Estrada & Rodríguez-Velázquez, 2005). $\mu_{j}(i)$ is the *i*-th of the *j*-th eigenvector and $\lambda_{j}$ is the eigenvalue corresponding to the *j-*th eigenvector. SC is categorized into mesoscale centrality.

$$\mathrm{SC}\left( i \right)=\sum_{j=1}^{N} {{[\mu}_{j}(i)]}^{2}sinh(\lambda_{j})$$

1. *Eigenvector Centrality (EC)*

The first eigenvector of the adjacency matrix is the one that corresponds to the largest eigenvalue and what we called EC. EC is a global centrality which captures the global features.

$$\mathrm{EC}\left( i \right)=\mu_{1}\left( i \right)=\frac{1}{\lambda_{1}}A\mu_{1}=\frac{1}{\lambda_{1}}\sum_{j=1}^{N} a_{ij}\mu_{1}(j)$$

1. *Page-rank Centrality (PC)*

PC is a variant of EC and thus a global centrality as well. It introduces a small probability (1-d=0.15) for random damping to handle walking traps on a graph (Boldi, Santini, & Vigna, 2009).

$$\mathrm{PC}\left( i \right)=r\left( i \right)=1-d+d\sum_{j=1}^{N} \frac{a_{ij}r(j)}{\sum_{i=1}^{N} a_{ij}}$$
